# Orbital malignant rhabdoid tumor with an aberrant immunophenotype: a case report

**DOI:** 10.1007/s12672-026-05397-z

**Published:** 2026-06-10

**Authors:** Hongqin Jia, Rongrong Cai, Yingwen Bi

**Affiliations:** Department of Pathology, Eye and ENT Hospital, Fudan University, 83 Fenyang Road, Shanghai, 200031 China

**Keywords:** Malignant rhabdoid tumor, Orbit, SMARCB1

## Abstract

**Background:**

Malignant rhabdoid tumor (MRT) is a highly aggressive pediatric neoplasm defined by SMARCB1/INI1 loss, and primary orbital involvement is rare.

**Case presentation:**

A 20-month-old girl presented with rapidly progressive left proptosis. Magnetic resonance imaging demonstrated a 2.1 × 1.3 × 1.6 cm temporal retrobulbar mass with focal bone erosion. The primary tumor was treated with piecemeal excision, followed by adjuvant chemoradiotherapy. Pathology showed large epithelioid tumor cells with rhabdoid morphology. Immunohistochemistry revealed diffuse expression of vimentin, CK, and CD34, but INI1 loss and SALL4 negativity. Fluorescence in situ hybridization confirmed a heterozygous SMARCB1 deletion. However, rapid local progression with extension into the temporal lobe occurred 3 months after completion of the treatment. Salvage therapy led to marked radiographic regression, but was discontinued due to severe cytopenias. The patient died of intracranial progression 34 months after the initial surgery.

**Conclusions:**

Orbital MRT can exhibit an atypical CD34-positive and SALL4-negative immunophenotype. The tumor demonstrates highly aggressive behavior, and adjuvant chemotherapy and radiotherapy may provide only temporary control.

## Background

Malignant rhabdoid tumor (MRT) is a highly aggressive soft tissue neoplasm, with a mortality rate of approximately 60%–80% [1, 2]. It is defined by rhabdoid morphology and the inactivation of genes encoding components of the SWI/SNF chromatin-remodeling complex, most commonly SMARCB1 and less frequently SMARCA4. MRT can arise in the central nervous system as atypical teratoid/rhabdoid tumor (AT/RT), in the kidney as rhabdoid tumor of the kidney (RTK), or in extrarenal soft tissues as extrarenal rhabdoid tumor (EMRT), accounting for approximately 30%, 25%, and 45% of cases, respectively [2]. Regardless of the primary site, MRTs share similar morphological and genetic features. Orbital MRT is exceptionally rare, with most reported cases occurring in young children. Here, we report a case of orbital MRT characterized by an atypical immunophenotype and orbital bone erosion.

## Case presentation

**Fig. 1:**
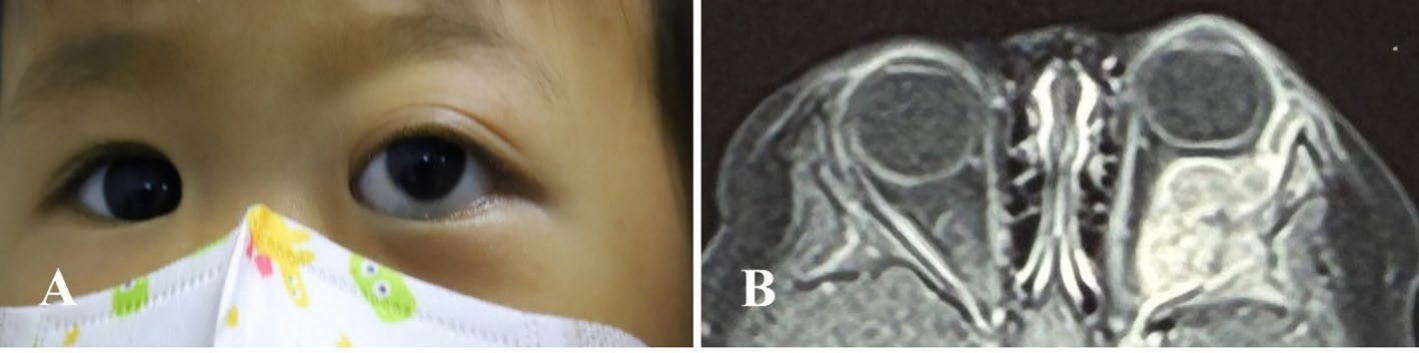
Initial appearance and radiographic findings. **A** Clinical photograph showing left proptosis. **B** Contrast-enhanced MRI demonstrating a retrobulbar mass in the left lateral orbit extending toward the orbital apex, with focal bone erosion

A 20-month-old girl was referred with a 1-month history of progressive left-sided proptosis (Fig. 1A). On examination, left upper eyelid ptosis was also noted. Formal visual acuity testing and dilated fundus examination could not be performed due to her young age. However, she was able to fixate and follow objects appropriately. Anterior segment examination was unremarkable bilaterally.

Orbital magnetic resonance imaging (MRI) performed at a referring hospital demonstrated a 2.1 × 1.3 × 1.6 cm mass in the left temporal retrobulbar orbit, with focal erosion of the lateral orbital wall (Fig. 1B). The patient subsequently underwent tumor excision via a lateral orbitotomy with lateral canthal extension. Intraoperatively, a large tumor was identified along the lateral orbital wall, with bone erosion and extension into the apex and inferior orbital fissure. Piecemeal excision of the mass was performed, followed by removal of the posterior lateral orbital wall.

**Fig. 2:**
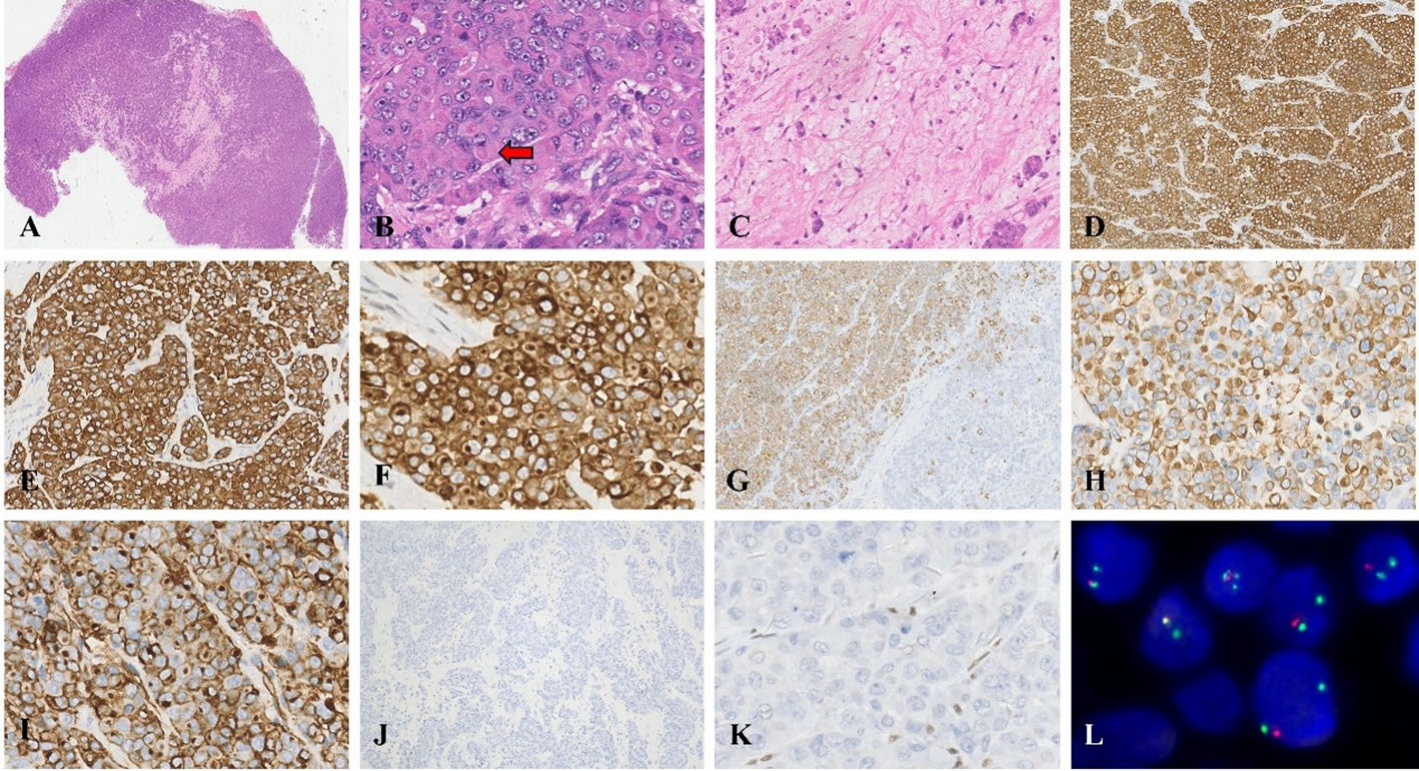
Histopathologic, immunohistochemical, and molecular findings. **A** Low-power view of tumor showing solid sheets and nests of tumor cells. **B** High magnification revealing epithelioid cells with eosinophilic paranuclear inclusions (arrow; ×400). **C** Scant and edematous stroma (×200). **D**–**G** Diffuse expression of CK (×100), CK8 (×200), EMA (×400), and focal CK20 positivity (×100), with EMA showing paranuclear dot-like staining. **H**, **I** Paranuclear dot-like staining of vimentin (×400) and CD34 (×400). **J** Negative SALL4 staining (×100). **K** Complete loss of INI1 expression (×400). **L** FISH analysis demonstrating one red and two green signals in tumor cell nuclei, consistent with heterozygous deletion of SMARCB1

Histopathology showed an unencapsulated tumor composed of solid sheets and nests of large epithelioid cells with abundant eosinophilic cytoplasm and eccentrically located nuclei with prominent nucleoli. Frequent mitotic figures were observed, and the stroma appeared edematous. Characteristic hyaline paranuclear inclusions were present, whereas necrosis was limited (Fig. 2A–C). Immunohistochemistry showed diffuse expression of epithelial markers (CK, CK8, and EMA), focal CK20 staining, and diffuse paranuclear dot-like staining for both vimentin and CD34 (Fig. 2D–I). The tumor was negative for desmin, SMA, MyoD1, myogenin, and SALL4 (Fig. 2J). Complete loss of nuclear INI1 (SMARCB1) expression was observed (Fig. 2K), and the Ki-67 proliferation index was approximately 50%. Fluorescence in situ hybridization (FISH) confirmed a heterozygous SMARCB1 deletion (Fig. 2L). This case was ultimately diagnosed as a malignant rhabdoid tumor. The diagnosis was confirmed by an independent external pathology consultation.

**Fig. 3:**
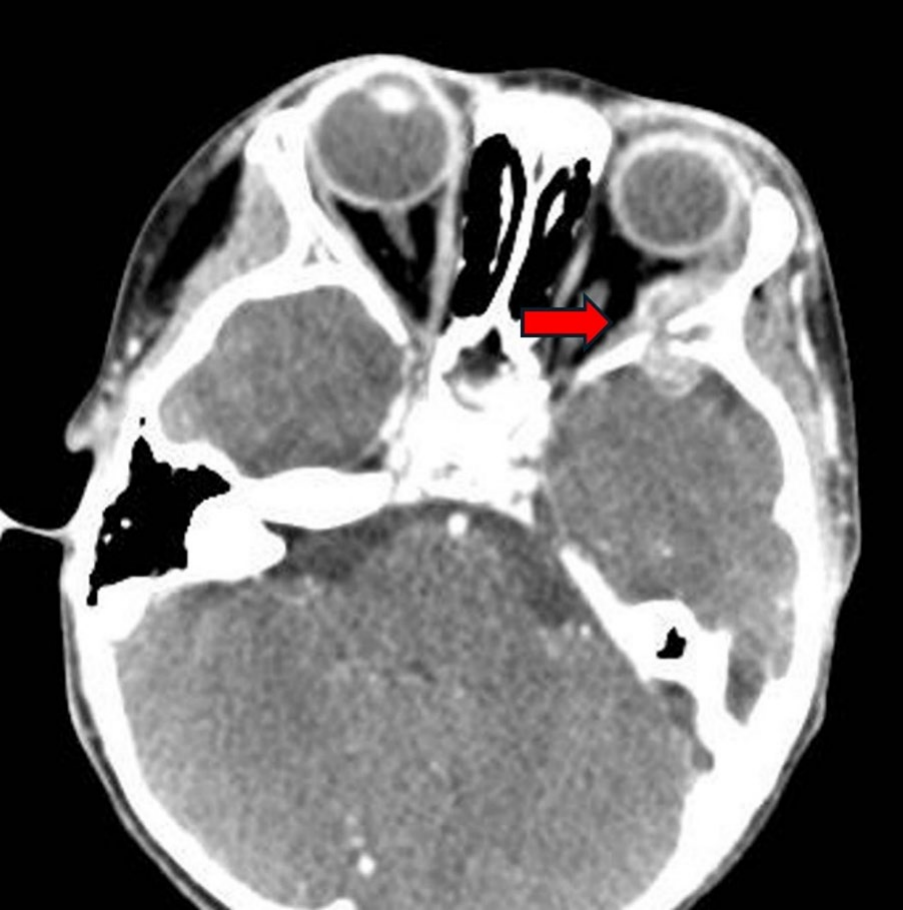
Radiographic findings of tumor progression. Contrast-enhanced CT performed 3 months after completion of adjuvant therapy showing progression of the lesion, with extension beyond the lateral wall into the left infratemporal fossa and the left temporal lobe (arrow)

Postoperative positron emission tomography–computed tomography (PET-CT) revealed no evidence of distant metastasis, but identified a 1.4 × 1.3 cm mildly FDG-avid lesion at the orbital floor, suspicious for residual disease. The patient received 12 cycles of chemotherapy administered in an alternating regimen: (i) vincristine, actinomycin D, and cyclophosphamide, with or without doxorubicin; (ii) vincristine, etoposide, and either ifosfamide or actinomycin D; and (iii) vincristine and irinotecan. Additionally, two cycles of vincristine and cyclophosphamide were administered concurrently with radiotherapy. Radiotherapy was delivered at a total dose of 64.8 Gy in 36 fractions. However, contrast-enhanced CT performed 3 months after completion of adjuvant therapy demonstrated progression of local residual disease extending beyond the lateral wall into the left infratemporal fossa and the left temporal lobe (Fig. 3). The patient subsequently underwent salvage therapy consisting of cisplatin and eribulin in combination with cabozantinib and sintilimab (a PD-1 inhibitor), achieving marked radiographic tumor regression. Chemotherapy and immunotherapy were maintained for approximately 12 months but were discontinued due to severe cytopenias. The patient ultimately died of intracranial progression 8 months after discontinuation of therapy.

## Discussion

Orbital MRT is extremely rare, with only 12 previously reported cases to date (Table 1) [3–14]. Most patients (9/12) presented at birth or during infancy. Compared with renal/intracranial (median age, 11–15 months) or extracranial (median age, 23–46 months) MRTs, orbital MRT appears to present at an earlier age [1, 15, 16]. In contrast, intraocular MRT may show a bimodal distribution, with two cases reported in infants and two in teenagers [17–20]. Despite their aggressive course, orbital MRTs rarely present with bone erosion or distant metastasis at diagnosis (Table 1), a frequency significantly lower than that observed in renal MRTs (reported metastasis rate, ~ 44%) and other extracranial MRTs (~ 24%) [1]. 

**Table 1:** Clinical features and outcomes of 13 orbital MRT cases

Case	Year (Author)	Age	Sex	Size (cm)	Surgery	Chemotherapy cycles	Radiotherapy	Follow-up	Invasion or met before first surgery	Rec/met	Outcome	IHC/molecular
1	Present case	20 mo	F	2.1 × 1.3 × 1.6	Partial excision	14	Y	34 mo	Bone erosion	Progression with intracranial extension	Died	IHC +: INI1 loss, Vimentin, CK, EMA, CK8, focal CK20, CD34 Molecular: SMARCB1 heterozygous deletion
2	2024 (Burgess) [3]	50 d	M	2.6 × 2.4 × 1.8	Biopsy and exenteration	10	N	11 mo	N	N	Alive	IHC +: INI1 loss, Vimentin, patchy CK, patchy EMA, CD56, SMA, focal NSE, focal CD99 Molecular: No SMARCB1 mutation detected
3	2023 (El Malih) [4]	1 mo	M	/	Biopsy	1	N	1 w after palliative chemotherapy	Contralateral cerebellar location	N	Died	IHC +: Vimentin, CK
4	2020 (Wilms) [5]	4 mo	/	2.5 × 2.1	Biopsy	2	Y	7 yr	N	N	Alive	IHC +: /
5	2020 (Biswas) [6]	1 mo	M	4.0 × 3.5	Biopsy and excision	4	Y	4 mo	N	Progression	Died	IHC +: INI1 loss, Vimentin, focal CK, focal EMA, focal SMA, focal GFAP
6	2014 (Mulay) [7]	6 w	M	/	Biopsy	0	N	Before adjuvant treatment	N	Met to intracranial	Died	IHC +: INI1 loss, Vimentin, CK, focal EMA, NSE, focal SMA
7	2013 (Karaman) [8]	8 w	F	2.8 × 2.2 × 1.8	Complete excision	3	N	3 mo	N	Rec	Died	IHC +: Vimentin, CK, EMA, focal S100
8	2009 (Kook)[9]	0	F	/	Biopsy and exenteration	1	N	3 mo	N	Met to the lung, liver, brain, and retroperitoneum	Died	IHC +: Vimentin, CK, EMA, CD99
9	2006 (Watanabe) [10]	15 mo	M	/	Partial excision	5	Y	4 yr	N	N	Alive	IHC +: INI1 loss, Vimentin, CK, EMA, SMA, CD99
10	1999 (Stidham) [11]	0	M	/	Biopsy with subtotal excision, second surgery	6	N	9 mo	Bone erosion, intracranial extension	N	Alive	IHC +: /
11	1998 (Gunduz)[12]	3 yr	F	/	Biopsy, exenteration (first relapse), and orbital debulking surgery with externalization of the maxillary sinus (second relapse)	6	Y	23 mo	N	Rec with maxillary sinus and intracranial extending	Died	IHC +: Vimentin, CK, EMA
12	1991 (Johnson) [13]	47 yr	M	3.2 × 2.2 × 1.1	Complete excision	0	Y	18 mo	N	N	Alive	IHC +: Vimentin
13	1989 (Rootman) [14]	6 w	M	/	Biopsy and evacuation with an ultrasonic suction fragmentation device	/	Y	24 mo	N	N	Alive	IHC +: Vimentin, CK (low molecular weight), EMA

Y, yes; N, no; F, female; M, male; mo, months; d, days; w, weeks; yr, years; Rec, recurrence; Met, metastasis; IHC: immunohistochemistry; /, not available

This rarity, together with overlapping morphologic and immunophenotypic features, contributes to diagnostic uncertainty and potential misclassification. Distinguishing MRT from epithelioid sarcoma (ES) can be particularly challenging, as both entities may exhibit epithelioid morphology and INI1 loss. While CD34 expression is classically associated with ES, its utility in differentiating these two tumors is limited, because CD34 positivity is reported in approximately 60% of MRTs and 81% of ES [21]. In contrast, SALL4 appears to be more specific, being positive in a higher proportion of MRTs and only rarely expressed in ES [21]. In our case, the young age at presentation, classic rhabdoid morphology, and INI1 loss supported MRT over ES despite the atypical profile of CD34 positivity and SALL4 negativity. These findings emphasize the need to integrate clinical context, morphology, and INI1/SMARCB1 status, and to pursue molecular confirmation where available, rather than relying on a limited immunopanel.

Although CD34 positivity and SALL4 negativity represent an aberrant immunophenotype in the present case, their prognostic significance in orbital MRT remains to be fully elucidated. SALL4, an embryonic stem cell regulator, has been implicated in tumor proliferation and progression, and its overexpression is generally associated with adverse outcomes in several malignancies [22, 23]. Conversely, the downregulation of SALL4 may be associated with loss of pluripotency and neuronal differentiation [24]. 

Regarding treatment, no standardized protocol currently exists for MRT. However, complete surgical resection has been associated with reduced mortality [1]. Most reported orbital cases present without distant metastasis and, in many instances, without bone erosion at diagnosis, supporting early and complete surgical excision as the cornerstone of local disease control [6]. The roles of radiotherapy and chemotherapy remain incompletely defined, although larger cohorts suggest that adjuvant radiotherapy may confer a survival benefit [1]. Notably, two orbital cases have achieved long-term disease control following proton therapy or Gamma Knife radiosurgery, indicating potential benefit in selected patients, while both cases presented without bone erosion [5, 6]. Complete histologic tumor necrosis after chemotherapy has been described in a case of orbital MRT, suggesting that at least a subset of orbital MRTs may be chemosensitive [6]. Recently, a prospective study demonstrated that an intensive UH-1 chemotherapy regimen, consisting of alternating three-drug combinations of vincristine/doxorubicin/cyclophosphamide and carboplatin/cyclophosphamide/etoposide, improved both event-free survival and overall survival in patients with MRT, supporting the potential benefit of aggressive treatment regimens in the management of this disease [25]. 

In the present case, the disease initially remained stable during multimodal therapy. However, progression of residual disease with intracranial extension occurred shortly after completion of the planned treatment course. This clinical course underscores the importance of complete excision, when feasible, and close radiographic surveillance.

This case report has some limitations. Germline testing for SMARCB1 and SMARCA4 alterations was not performed, meaning an underlying genetic predisposition cannot be entirely ruled out. Furthermore, the original postoperative PET-CT images were unavailable for inclusion in this report.

In conclusion, our case suggests that orbital MRT may be considered in the differential diagnosis of young children presenting with rhabdoid morphology and INI1 loss, even in the presence of an aberrant immunophenotype (including CD34 positivity and SALL4 negativity). Given the aggressive nature and poor prognosis of this disease, more effective treatment strategies remain urgently needed.”

## Data Availability

The data analyzed in this case are not publicly available due to patient privacy and ethical restrictions but are available from the corresponding author on reasonable request.

## Abbreviations

ES: Epithelioid sarcoma
FISH: Fluorescence in situ hybridization
MRI: Magnetic resonance imaging
MRT: Malignant rhabdoid tumor
PET-CT: Postoperative positron emission tomography–computed tomography
